# Use of Multimodal Therapies to Treat Severe Orthostatic Hypotension From Autonomic Failure

**DOI:** 10.7759/cureus.31990

**Published:** 2022-11-28

**Authors:** Teja Chakrala, Anshul Jain, Kun Xiang, Richard Kerensky

**Affiliations:** 1 Internal Medicine, University of Florida College of Medicine, Gainesville, USA; 2 Cardiology, University of Florida College of Medicine, Gainesville, USA

**Keywords:** multimodal therapy, dizziness, orthostatic, hypotension, syncope

## Abstract

Orthostatic hypotension is one of the most debilitating features of autonomic failure. Presentations tend to be diverse and non-specific, ranging from dizziness and lightheadedness to loss of consciousness. Early recognition of this illness may prove to be difficult, and control of symptoms typically requires a multidisciplinary approach with patient education, pharmacologic, and device therapies. This condition tends to be associated with significant patient morbidity and poor quality of life, and treatment regimens typically require frequent adjustments before optimal symptom control is achieved. However, with a multidisciplinary approach, utilization of multimodal therapies, and proper identification of underlying pathophysiologic mechanisms, symptom improvement may be achieved.

An 81-year-old male was admitted to our hospital following a syncopal episode. While hospitalized, he experienced severe syncope and was treated with physical therapy, midodrine, fludrocortisone, octreotide, erythropoietin, and intravenous iron infusions. He displayed minimal improvement and then had a biventricular pacemaker placed and had a resolution of all postural symptoms.

## Introduction

Orthostatic hypotension (OH) is the second most common cause of syncope and causes significant disability in affected patients [1,2]. It is prevalent in the elderly and occurs secondary to conditions causing autonomic failures such as neurodegenerative disorders and autonomic neuropathies [2]. It tends to be associated with diverse clinical presentations with non-specific symptoms such as dizziness, headaches, and lightheadedness, making its early recognition difficult [3]. Diagnosis of the condition is made when there is a sustained reduction of at least 20 mm Hg of systolic blood pressure or 10 mm Hg of diastolic blood pressure within 3 minutes of standing. Management includes a combination of both pharmacologic and non-pharmacologic strategies that are frequently found to be unsatisfactory [1-3]. Initial strategies should focus on reversible factors such as culprit medications, treatment of comorbidities, and patient education on conservative therapies [3-5]. In any case, treatment goals should be individually tailored to patients and may require multiple pharmacologic treatments to achieve symptomatic relief [5]. Herein, we present a case of an 81-year-old male with ischemic cardiomyopathy who presented to our hospital with syncope and required a multimodal treatment regimen to treat his severe symptomatic OH.

## Case presentation

An 81-year-old male with a past medical history of chronic systolic heart failure with an ejection fraction of 20%, secondary to ischemic cardiomyopathy, four-vessel coronary artery bypass grafting (1999), left ventricle (LV) thrombus on anticoagulation, type 2 diabetes mellitus, and a recent right middle cerebral artery stroke was admitted to our hospital for evaluation of syncope. The patient was on his way to a routine cardiology appointment when he experienced a syncopal event in the parking lot and was subsequently transported to our emergency department. Because the patient had no recollection of the event, history was obtained from his wife who stated he was exiting his vehicle when he began to experience lightheadedness and dizziness following which he lost consciousness and fell backward, hitting his head on the ground. The patient had reported not having experienced similar episodes in the past. Upon presentation, his examination was significant for positive orthostatic vital signs with blood pressure decreasing from 112/74 mmHg to 83/49 mmHg upon standing. Throughout his hospital course, he continued to experience recurrent syncopal episodes so severe that he would develop dizziness and lightheadedness within five seconds of sitting upright from a supine position. On one occasion, while working with physical therapy (PT), he lost consciousness about 10 seconds after standing from a seated position and regained consciousness after laying supine.

**Figure 1:**
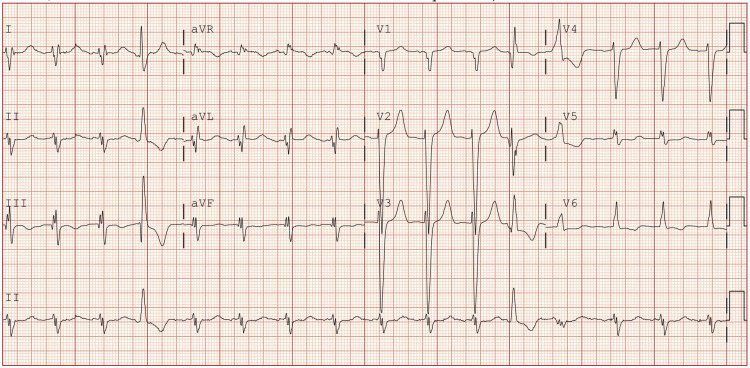
EKG on admission

Serum bloodwork revealed chronic normocytic anemia, serum creatinine of 1.93 mg/dL (elevated from patients’ baseline of 1.3 mg/dL), brain natriuretic peptide level of 614 pg/mL (decreased from 1,096 pg/mL several months prior), and high sensitivity troponin-I levels which were unremarkable. Additional laboratory work showed a clean urinalysis and a negative COVID-19 test. A chest x-ray showed findings of cardiomegaly without pleural effusions or signs of an infectious process, and computed tomography imaging of the head showed soft tissue swelling over the posterior scalp, moderate cerebral atrophy, and ischemic disease. The electrocardiogram showed a left bundle branch block (LBBB) and QRS duration of 150ms (Figure 1). Transthoracic echocardiography showed severely reduced LV systolic function with an ejection fraction of 15%-20% and severe diffuse hypokinesis of the LV walls. Cerebral angiography was performed to assess for vertebrobasilar insufficiency which showed 62% stenosis of the left internal carotid artery however nothing is amenable to treatment. Transcranial Doppler was performed which did not demonstrate abnormalities in vertebral artery flow. Cosyntropin stimulation testing to assess for adrenal insufficiency was also negative.

**Figure 2:**
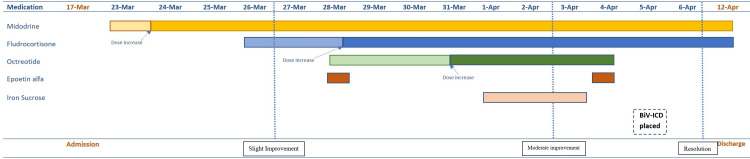
Timeline of medications

Treatment was initiated with compression stockings and intermittent intravenous fluid boluses totaling 1.75 liters over three days. He remained unable to participate with PT due to ongoing syncope. Six days after being admitted, he was started on midodrine 5mg three times daily which was increased to 10mg three times daily the following day. Fludrocortisone was initiated at 0.1mg daily and then increased to 0.3mg several days later. He continued to endorse dizziness, however, was able to sit upright for about one minute before symptom onset. Subcutaneous (SC) epoetin alfa 10,000 units weekly and SC octreotide 100mcg three times daily were started, and intravenous iron sucrose was added several days later. Following these medication changes, the patient demonstrated significant improvement in his symptoms. He was able to participate with PT and displayed a lower premature ventricular complex burden on telemetry. Although he displayed symptom improvement, his orthostatic vitals remained positive, and continued to exhibit trouble standing. Given his advanced heart failure, LBBB, and wide QRS duration, he underwent biventricular-implantable cardioverter defibrillator (BiV-ICD) placement. Several days later, he was able to ambulate to a chair without assistance and had a complete resolution of his symptoms. He was subsequently discharged home on midodrine and fludrocortisone. (Pharmacologic agent initiation corresponding with symptom response is demonstrated in Figure 2.) On follow-up in the clinic two weeks later, the patient was able to stand multiple times without dizziness or unsteadiness and continued to deny symptoms.

## Discussion

Orthostatic intolerance is a term that refers to an abnormal response of the autonomic nervous system (ANS) causing patients to develop symptoms when in an upright posture [1]. Syndromes of orthostatic intolerance can be classified into OH, neurally mediated syncope, and postural tachycardia syndrome [2]. OH can be further subdivided into two categories based on etiopathogenesis: neurogenic OH, referring to structural ANS failure, and non-neurogenic OH, or functional ANS failure [1,2].

Neurogenic OH (nOH) results primarily from inadequate norepinephrine release secondary to autonomic failure [3]. It can occur as a manifestation of primary neurodegenerative disorders such as Parkinson’s disease, Lewy body dementia, and multiple system atrophy. Secondary etiologies include diabetes mellitus, drug-related causes, cardiovascular disease, and renal failure [3,4]. Patients with OH can present with a myriad of symptoms or none at all. When present, patients report dizziness, lightheadedness, fatigue, blurry vision, difficulty concentrating, or feeling faint [3,5]. Symptoms tend to be worse in the early morning, likely as a result of the nocturnal polyuria that occurs in these patients. Symptoms worsening after meals, with physical activity, and a rise in body temperature, tends to occur, due to cerebral hypoperfusion [4,5]. A diagnosis of OH is made when there is a reduction of systolic blood pressure of at least 20 mmHg or diastolic blood pressure of at least 10 mmHg within 3 minutes of standing from a seated position [4]. Patients with OH are at increased risk of complications including frequent falls, cognitive impairment, and dementia [4]. This is thought to be due to repeated episodes of hypotension resulting in cerebral hypoperfusion and consequential neuronal injury. One study found that OH was a strong predictor of future cardiovascular events including myocardial infarction, stroke, and death [6].

To better guide therapeutic strategies, a stepwise approach has been recommended in addition to the utilization of clinically proven grading scales such as the Orthostatic Hypotension Questionnaire (OHQ) [5]. Prior to treatment, a comprehensive medication review is essential to identify medications that may exacerbate orthostatic symptoms [7,8]. Dose reduction and discontinuation of diuretics, vasodilators, and negative chronotropic agents such as beta-blockers are imperative. Ensuring adequate hydration by encouraging generous fluid intake is key to achieving intravascular volume repletion [8]. Salt intake is also recommended, however, should be closely monitored in patients with heart failure or severe congestion. Compression garments and physical conditioning, particularly of the lower extremities, are key to preventing venous pooling and improving venous return to the heart [3,8].

Pharmacologic measures are initiated when these strategies fail to improve symptoms [9]. Many therapeutic options are available, however lack of large-scale clinical trials and the presence of significant adverse reactions to these drugs have contributed to challenges in treatment. An individualized regimen should be tailored to each patient, taking into consideration symptom severity, co-morbidities, and treatment risks [5,9]. The provision of patient education on how to measure blood pressure throughout the day is paramount [3,9]. Two drugs have been approved by the US Food and Drug Administration for the treatment of symptomatic nOH: the alpha-adrenergic agonist midodrine and the norepinephrine precursor droxidopa [3,9]. The latter has been found to improve nearly all nOH symptom scores versus placebo and is believed to increase blood pressure by acting on the neurovascular junction to increase vascular tone [9]. Fludrocortisone, a synthetic mineralocorticoid, is widely used off-label but can lead to supine hypertension [5,8]. This medication primarily acts by promoting intravascular volume expansion and subsequent symptom relief [4,9]. Adjunctive therapies have also been trialed in patients with varying efficacies. Recombinant human erythropoietin, in the form of epoetin alfa, increases standing blood pressure and improves orthostatic tolerance for patients with anemia that often occurs with autonomic failure [5]. This is thought to occur by increasing the red cell mass and central blood volume. Iron supplementation is usually required, particularly during the period when the hematocrit is increasing. Somatostatin analogs, such as octreotide, attenuate the gastrointestinal hormonal response to food ingestion by inhibiting the vasoactive peptide release [9]. They also increase cardiac output and splanchnic vascular resistance, preventing the pooling of blood in the gut. The net effect is attenuation of the fall in the postprandial blood pressure in patients with autonomic failure.

Successful treatment of severe OH associated with chronotropic incompetence, by means of cardiac tachypacing, has been reported in the literature [10]. The mechanism by which this occurs is thought to be due to the resulting increase in inotropic stimulus with an increase in stroke volume, contraction velocity, and venous return to the heart following device placement [10]. There is limited data overall regarding the role of pacemakers and cardiac resynchronization therapy in OH. However, if chronotropic insufficiency or LV failure worsen OH symptoms, especially in elderly patients, device therapy can be considered [3,7]. A decrease in OH symptoms when using a closed-loop stimulation rate-adaptive sensors has been studied where activities requiring a lower energy expenditure, such as standing, are more dramatically affected by improvements in the chronotropic cardiac response [4,10].

## Conclusions

OH can prove to be a debilitating condition if not adequately treated. A multidisciplinary, patient-centered approach is crucial in its management with a focus made on patient education. Initial emphasis should be made on non-pharmacologic strategies such as compression garments and physical conditioning along with an increased intake of fluids and existing medication adjustments. However, most patients may end up requiring pharmacologic therapies for symptom relief. Multimodal regimens may become necessary for the treatment of patients with severe presentations and characterizing symptom improvement through the utilization of validated questionnaires such as the OHQ may prove beneficial in tailoring medication regimens. Future multicenter studies with large patient populations and longer follow-ups will help further guide treatment strategies.
